# Dewey’s Orthodontia

**Published:** 1914-12-15

**Authors:** 


### BIBLIOGRAPHY.

DEWEY'S ORTHODONTIA. A new book on orthodontia by Martin Dewey, D.D.S., M.D., has just been published by the C. V. Mosby Co., of St. Louis. The title to this work is "*Practical Orthodontia,*" and that idea seems to prevail throughout the book, although the theory of classification by Angle is kept to the fore all the way through the book, as well as all other scientific facts that have established orthodontic treatment as a practicable theory. The author is a practicing orthodontist, conducts a training school in orthodontia and is to be the editor of a journal devoted exclusively to the treatment of orthodontia, and he has also had much experience as a teacher, therefore he ought to be in a position to write good material for students of this subject. He has written a valuable book from the technical view point and we have no doubt it will meet a great need as a brief text for students use, as its emphysis is largely placed on technical execution. For this reason it will also appeal to the practitioner who is endeavoring to carry this kind of work in a general practice. While the author has followed in the main the methods and appliances of the Angle school, he has made use of many valuable methods of the other so-called schools. It is fully illustrated with many original cuts, and the text is well composed. It is a book of

342 pages and may be had of the dental supply houses, at $3.50.

